# Intrapancreatic recurrence of intraductal tubulopapillary neoplasm (ITPN) 16 years after the initial surgery for noninvasive ITPN: a case report

**DOI:** 10.1186/s40792-018-0497-1

**Published:** 2018-08-16

**Authors:** Kiyoshi Saeki, Yoshihiro Miyasaka, Yoshihiro Ohishi, Takeo Yamamoto, Ryota Matsuda, Naoki Mochidome, Yasuhisa Mori, Kohei Nakata, Takao Ohtsuka, Kousei Ishigami, Yosuke Minoda, Yutaka Koga, Yoshinao Oda, Masafumi Nakamura

**Affiliations:** 1 Department of Surgery and Oncology, Graduate School of Medical Sciences, Kyushu University, Fukuoka, Japan; 2 Department of Anatomical Pathology, Graduate School of Medical Sciences, Kyushu University, Fukuoka, Japan; 3 Department of Clinical Radiology, Graduate School of Medical Sciences, Kyushu University, Fukuoka, Japan; 4 Department of Medicine and Bioregulatory Science, Graduate School of Medical Sciences, Kyushu University, Fukuoka, Japan

**Keywords:** Intraductal tubulopapillary neoplasm, Pancreas, Intrapancreatic recurrence

## Abstract

**Background:**

Intraductal tubulopapillary neoplasm (ITPN) is a rare pancreatic intraductal neoplasm. It is characterized by a tubulopapillary growth pattern, entirely high-grade atypical cells, minimal cytoplasmic mucin, and no obvious luminal mucin secretion. Most of its biological nature remains unclear.

**Case presentation:**

We herein report a case of intrapancreatic recurrence of ITPN in the remnant pancreas of a patient who underwent pancreatoduodenectomy 16 years previously for a noninvasive intraductal pancreatic head tumor. We reexamined the primary tumor and compared it with the most recently resected specimen. Histologically, the primary tumor showed a tubulopapillary growth of high-grade atypical cells with scanty cytoplasmic mucin, which was similar to the recently resected specimen except for the invasive area. Immunohistochemically, the neoplastic cells in both specimens showed focal staining of MUC1 and positivity for MUC6 but negativity for MUC2, MUC5AC, CDX2, and trypsin. Molecular analysis revealed no KRAS/GNAS/BRAF/PIK3CA mutations in either of the specimens.

**Conclusions:**

These findings of the original tumor and recently resected tumor were compatible with the features of ITPN. Thus, recurrence is possible even for a primary noninvasive ITPN, and long-term surveillance is recommended.

## Background

Intraductal tubulopapillary neoplasm (ITPN) is a rare neoplasm accounting for < 1% of all exocrine neoplasms of the pancreas [1, 2]. According to the 2010 World Health Organization (WHO) classification [1], pancreatic intraductal neoplasms are divided into intraductal papillary mucinous neoplasm (IPMN) and ITPN. Both IPMN and ITPN are recognized precursors for pancreatic cancer. ITPN is distinguishable from IPMN by its tubulopapillary growth pattern with entirely high-grade atypical cells and no obvious mucin secretion. In addition, the immunohistochemical mucin core protein expression patterns and molecular alterations of ITPN are distinct from IPMN.

Intrapancreatic recurrence sometimes occurs after surgery for pancreatic cancer [3, 4]. Although intrapancreatic recurrence of noninvasive pancreatic lesions, such as IPMN, has been reported [5], it is extremely rare. In addition, recurrent lesions usually arise within a few years after the previous surgery. We herein report a rare case of intrapancreatic recurrence of ITPN that occurred as long as 16 years after the initial surgery for noninvasive ITPN.

## Case presentation

**Fig. 1:**
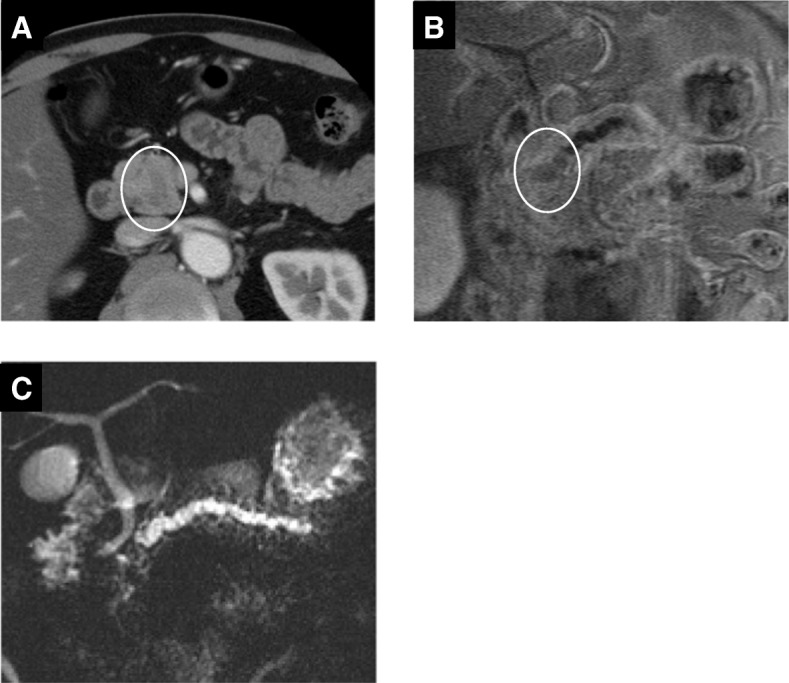
Preoperative imaging findings during the assessment of the initial resected pancreas head lesion. **a** Enhanced computed tomography showed a dilated main pancreatic duct (MPD) with an enhancing mass (20 mm in diameter) at the head of the pancreas (circle). **b**, **c** Magnetic resonance cholangiopancreatography showed a low-intensity area in the pancreas head (circle) and dilation of the distal side of the MPD (5 mm in diameter)

**Fig. 2:**
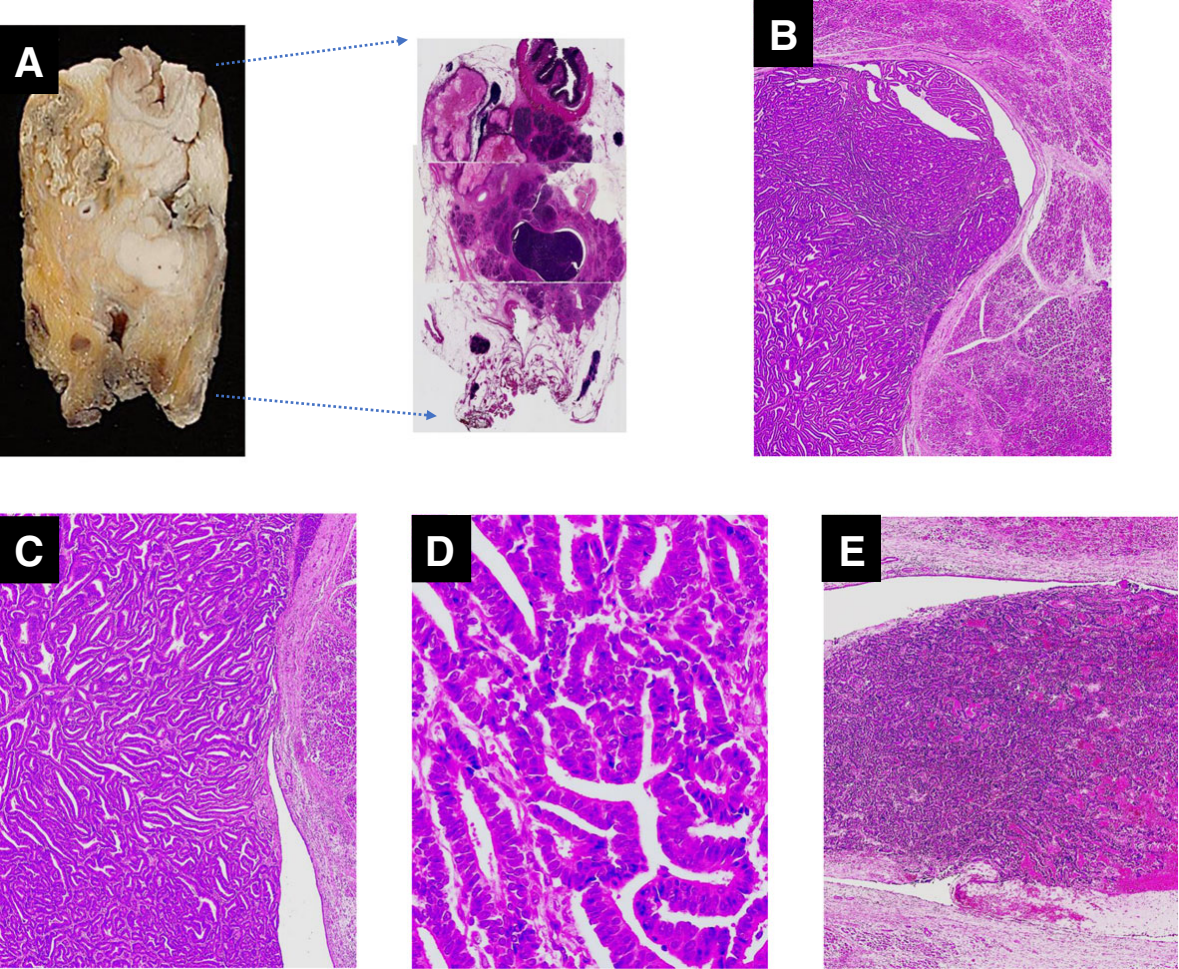
Pathological findings of the initial resected specimen of the pancreas head lesion. **a** Macroscopic findings of the resected specimen. The tumor appeared as a solid nodule with a dilated main pancreatic duct. Secreted mucin was not visible. **b**–**e** Microscopic findings of the resected specimen. **b** Microscopic examination demonstrated that the tumor was confined to the pancreatic duct, and no apparent invasive carcinoma components were observed (original magnification, × 20). **c** Microscopic findings demonstrated a tubulopapillary growth pattern with scanty cytoplasmic mucin (original magnification, × 40). **d** The neoplastic cells showed a uniform high-grade atypia (original magnification, × 400). **e** Necrotic tissue within the tumor was observed (original magnification, × 40)

A 54-year-old man was referred to our institution because of ultrasonography findings of a hypoechoic pancreatic head mass with a dilated main pancreatic duct (MPD). Blood tests showed elevated liver enzymes and normal tumor marker levels: glutamic oxaloacetic transaminase, 47 U/L (reference range at our institution, 13–33 U/L); glutamic pyruvate transaminase, 81 U/L (6–30 U/L); γ-glutamyl transpeptidase, 135 U/L (10–47 U/L); carcinoembryonic antigen, 1.0 ng/ml (0–3.2 ng/ml); and carbohydrate antigen 19–9, 10.1 U/ml (0–37.0 U/ml). Enhanced computed tomography (CT) revealed a dilated MPD with a 20-mm-diameter enhancing mass at the head of the pancreas (Fig. 1a). Magnetic resonance cholangiopancreatography showed a low-intensity area in the pancreatic head and dilation of the distal side of the MPD (5 mm in diameter) (Fig. 1b, c). Duodenoscopy showed a normal appearance of the orifice of the major papilla, while endoscopic retrograde pancreatography revealed a complete obstruction of the MPD at the area of the pancreatic head. Although pancreatic juice cytology was negative for malignancy, the pancreatic head mass was still highly suspicious of cancer based on the imaging findings. The patient subsequently underwent pancreatoduodenectomy. On gross examination of the resected specimen, the tumor appeared as a solid nodule with a dilated MPD and no visible mucin (Fig. 2a). On microscopic examination, the tumor showed a tubulopapillary growth pattern with scanty cytoplasmic mucin (Fig. 2b, c). The tumor was confined to the pancreatic duct; we observed no apparent invasive carcinoma component consisting of individual cells or small, angulated nonmucinous glands extending away from the periphery of the involved ducts into the surrounding desmoplastic stroma. The neoplastic cells showed a uniform high-grade atypia (Fig. 2d). Necrotic tissue was also seen (Fig. 2e). All surgical margins were negative. Immunohistochemical assessment of mucin core protein expression in the neoplastic cells showed focal staining of MUC1, positivity for MUC6, and negativity for MUC2 and MUC5AC. The neoplastic cells were immunohistochemically positive for cytokeratin 7 (CK7) and CK19 but negative for CDX2 and trypsin. The Ki-67 labeling index was 20%. The pathological diagnosis of the resected specimen at that time was an intraductal papillary and tubular tumor with severe atypia of the pancreatic head. Thereafter, clinical surveillance by blood testing was performed every 2 months for 6 years. During the follow-up, the carcinoembryonic antigen level steadily increased up to 7.0 ng/ml (reference range, 0–3.2 ng/ml); thus, additional surveillance was implemented using alternate CT and MRI examinations every 6 months.

**Fig. 3:**
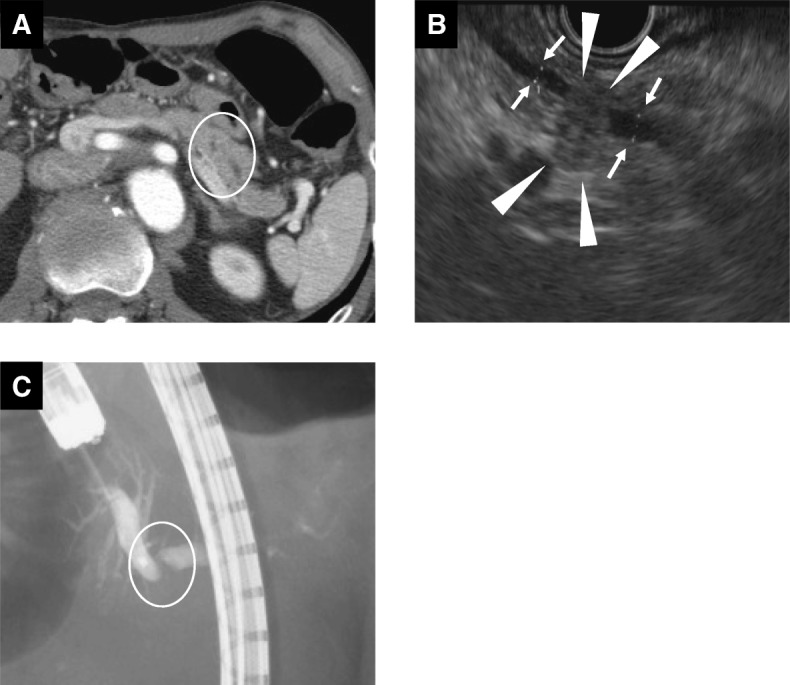
Preoperative imaging findings during the assessment of the resected remnant pancreas lesion 16 years after the initial operation. **a** Enhanced computed tomography showed a low-density mass at the remnant pancreatic body (circle). **b** Endoscopic ultrasonography showed an isoechoic and hypovascular mass (7 mm in diameter) protruding into the main pancreatic duct (MPD) of the pancreas body (arrowhead). A slight change in the diameter of the MPD between the proximal side and the distal side of the mass was observed (arrow). The MPD wall adjacent to the mass was ill-defined. **c** Endoscopic retrograde pancreatography showed a localized narrow segment with irregular tapering (4 mm in length) in the MPD of the remnant pancreatic body (circle)

**Fig. 4:**
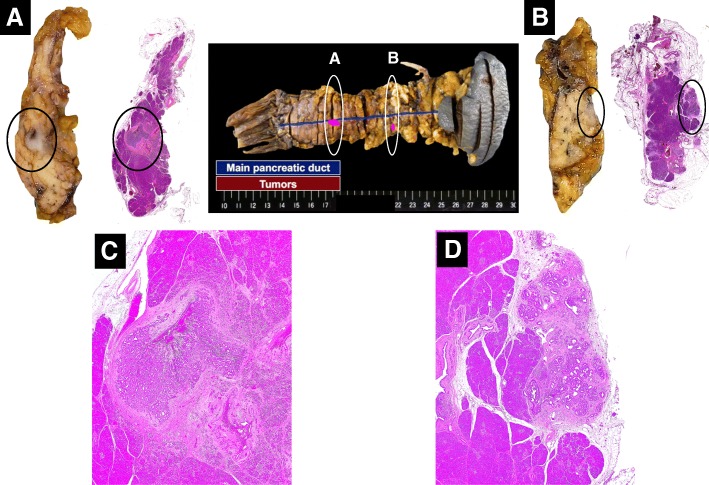
Pathological findings of the secondary resected specimen of the pancreas body and tail lesions. **a**, **b** Two tumors were identified at the pancreas **a** body and **b** tail. **a** A white solid mass (5 mm in diameter) with well-defined margins adjacent to the main pancreatic duct (MPD) was observed in the pancreas body (circle). **b** A white solid mass (5 mm in diameter) with ill-defined margins farther away from the MPD was also observed in the pancreas tail (circle); this mass had not been detected in the preoperative images. Neither of these tumors exhibited luminal mucin secretion. **c**, **d** Microscopic findings of the resected specimen (**c** pancreas body lesion, **d** pancreas tail lesion). **c** A solid nodular tumor with a dilated MPD was observed (original magnification, × 20). **d** The tumor with ill-defined margins was farther away from the MPD (original magnification, × 20)

**Fig. 5:**
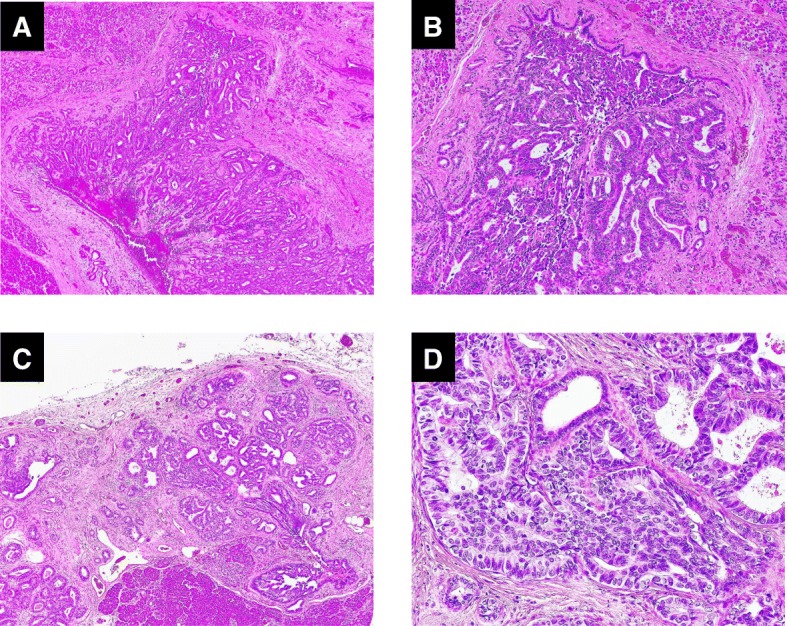
Pathological findings of the secondary resected specimen of the pancreas body and tail lesions. **a**–**d** Microscopic findings of the resected specimen (**a**, **b** pancreas body lesion; **c**, **d** pancreas tail lesion). **a** The tumor extended around and into the MPD (original magnification × 40). **b** The tumor showed a tubular growth pattern with scanty cytoplasmic mucin (original magnification × 100). **c** The tumor showed a tubular growth pattern (original magnification × 40). **d** The neoplastic cells showed a uniformly high-grade atypia, and apparent invasive carcinoma lesion was present (original magnification × 200)

Sixteen years after the operation, enhanced CT showed a low-density mass at the remnant pancreatic body (Fig. 3a). Endoscopic ultrasonography demonstrated a 7-mm-diameter isoechoic and hypovascular mass protruding into the MPD of the pancreatic body with a slight dilation of the MPD of the distal side of the mass. Additionally, the MPD wall adjacent to the mass was ill-defined (Fig. 3b). Endoscopic retrograde pancreatography exhibited a localized narrow segment with irregular tapering of 4 mm in length in the MPD of the remnant pancreatic body (Fig. 3c). Pancreatic juice cytology was positive for malignancy. The patient was diagnosed with remnant pancreatic cancer and subsequently underwent completion pancreatectomy. On gross examination of the resected specimen, two tumors were identified at the pancreatic body and tail (Fig. 4a, b). The lesion at the pancreatic body was a 5-mm-diameter white solid mass with well-defined margins adjacent to the MPD (Figs. 4a, 5a, b). The other lesion, which had not been detected preoperatively by the imaging studies, was located at the pancreatic tail and also presented as a 5-mm-diameter white solid mass but had unclear margins and was apart from the MPD (Figs. 4b, 5c, d). Neither of these tumors exhibited luminal mucin secretion. On microscopic examination, the remnant tumors in the body (Fig. 4c) and tail (Fig. 4d) were morphologically very similar to the original pancreatic head lesion, while the presence of invasive carcinoma was confirmed in both the remnant tumors. The immunohistochemical results of the remnant tumors were identical to those of the original lesion.

Moreover, we investigated the KRAS/BRAF/GNAS/PIK3CA mutational status (mutational analysis for exon 2 of KRAS, exon 15 of BRAF, exons 9 and 20 of PIK3CA, and exons 8 and 9 of GNAS) of the original tumor (pancreatic head) and the recently resected tumors from the pancreatic body and tail. These analyses revealed no KRAS/BRAF/GNAS/PIK3CA mutations in all tumor samples. We also reexamined the previous pancreatic head tumor, and the findings were compatible with ITPN according to the current 2010 WHO criteria [1]. Based on the morphological and biological similarities between the original tumor in the head and the recent tumors from the body and tail, we diagnosed the two lesions as intrapancreatic recurrent ITPNs from the original pancreatic head tumor.

The patient underwent postoperative adjuvant chemotherapy using S-1 (tegafur-gimeracil-oteracil potassium). At the time of this writing, he was alive with no evidence of disease during a follow-up period of 9 months after remnant pancreatectomy.

### Discussion

ITPN was first designated by Yamaguchi et al. [2] and was categorized as a new entity in the pancreatic intraductal neoplasm family in the 2010 WHO classification of tumors of the digestive system [1]. Characteristically, these pancreatic intraductal neoplasms show a tubulopapillary growth pattern with entirely high-grade atypical cells and have less cytoplasmic mucin and no obvious luminal mucin secretion. Histologically and biologically, ITPN can be distinguished from other pancreatic intraductal neoplasms such as conventional pancreatic ductal adenocarcinoma (PDAC), pancreatic intraepithelial neoplasms (PanIN), IPMN, and intraductal variant of acinar cell carcinoma (ACC).

One of the characteristics of ITPN is the appearance of a solid nodular tumor obstructing the dilated ducts on macroscopic examination [2]. In both the initial pancreas head lesion and the remnant pancreas body lesion, we observed a solid nodular tumor with a dilated MPD on macroscopic examination. The pancreatic tail lesion was located apart from the MPD, and we did not observe a solid nodular tumor obstructing dilated ducts on macroscopic examination. However, microscopic examination showed a tubulopapillary growth with entirely high-grade atypical cells, minimal cytoplasmic mucin, and no visible luminal mucin secretion, which was identical to the histological features of ITPN in both the primary tumor and the pancreatic body tumor. Additionally, all immunohistochemical and molecular results were identical to both the primary tumor and the pancreatic body tumor. Therefore, the pancreatic tail tumor was also thought to be ITPN.

Although the neoplastic cells in IPMN usually express MUC5AC in all its subtypes (gastric, intestinal, pancreatobiliary, and oncocytic) [6], MUC5AC expression is negative in ITPN [2, 7–12]. The expression of MUC2 and CDX2, which is a characteristic of the intestinal lineage [13, 14], is also absent in ITPN [2, 7–12]. However, MUC1 and MUC6 show positive expression in most cases of ITPN [2, 7–12]. CK7 and CK19 expression are measured to assess ductal differentiation, and CK7, CK19, or both are reportedly strongly positive in all patients with ITPN [2, 7]. Additionally, the finding of intraductal lesions with a tubulopapillary growth pattern and absence of KRAS mutations also suggests the possibility of an intraductal variant of ACC [15, 16], which would show positive immunohistochemical staining for trypsin.

We analyzed the three tumors using immunohistochemical staining in the present case. All three lesions in this case were negative for MUC2, MUC5AC, and CDX2 expression, and the neoplastic cells of the three lesions showed focal staining for MUC1 and positive staining for MUC6, CK7, and CK19 expression. The possibility of an intraductal variant of ACC was easily ruled out by the negative staining for trypsin, which was also compatible with all three lesions. Thus, the immunohistochemical findings are in accordance with our diagnosis that the original tumor was indeed ITPN and that the remnant pancreatic tumors were recurrences of the initial tumor.

More than 90% of PDACs harbor KRAS mutations, and some proportion of PDACs without KRAS mutations also harbors BRAF mutations [17–19]. KRAS mutations are also frequently seen in PanIN and IPMN [20–22]. About 40 to 60% of IPMNs reportedly harbor GNAS mutations alone, and the vast majority of IPMNs harbor KRAS and/or GNAS mutations [21–23]. Conversely, ITPN lacks KRAS/GNAS/BRAF mutations [2, 24–26]. The three lesions in this study were genetically consistent with these characteristics, whereas none of the lesions harbored any KRAS/GNAS/BRAF mutations. Some studies have shown that ITPN can also be linked to PIK3CA mutations [24–26], but this was not observed in the present case. However, PIK3CA mutations occur in less than 30% of cases; thus, ITPN is still highly probable despite its absence in our samples. These genetic findings also support the probable diagnosis of ITPN rather than IPMN or conventional PDAC.

The remnant pancreatic tumor was identified by enhanced CT 16 years after the initial operation. Enhanced CT 6 months before the confirmed diagnosis of ITPN showed no mass at the remnant pancreas. Considering that the ITPN tumor grew from undetectable to 7 mm within 6 months despite the lack of tumor development for 16 years after the initial surgery, the growth rate of ITPN might be relatively rapid once the tumor has become visible.

ITPN is a rare tumor, and data obtained from clinical surveillance are very limited [2, 7–12, 26]. Although ITPN with an associated invasive carcinoma has a poorer prognosis than noninvasive ITPN, the clinical course of ITPN is relatively indolent compared with conventional PDAC, even with the presence of invasive carcinoma [7]. To date, six cases of local recurrence of ITPN have been reported in the English-language literature [2, 7, 12]; two recurrences were observed at 12 and 34 months postoperatively, while the other four cases lacked timeline information [2, 7, 12]. Additionally, some studies have shown that recurrence of ITPN can occur even if no identifiable invasive carcinoma was present in the initial lesion [2, 7]. Yamaguchi et al. reported a case of intrapancreatic recurrence 12 months after the initial operation. In their case, the original lesion was noninvasive ITPN, and the morphological and molecular features of the recurring neoplasm were identical to those of the original lesion [2]. In the present case, invasive carcinoma was not identified in the original ITPN of the pancreatic head. However, intrapancreatic recurrence as invasive carcinoma occurred on the remnant pancreas 16 years after the initial surgery. Due to the limited numbers of long-term studies on ITPN and its recurrence rates, further investigation is needed.

Studies of remnant pancreatic lesions after resection of IPMN have been reported [27–30]. Similar to IPMN, three possible mechanisms of development of remnant pancreatic lesions after resection of ITPN can be suggested: (1) the presence of residual microscopic neoplastic cells at the resected margin in the remnant pancreas, (2) intraductal or intrapancreatic lymphovascular spread to the remnant pancreas, and (3) metachronous, multicentric development. In our case, the surgical margin at the time of pancreatoduodenectomy was negative for neoplastic cells, and the remnant pancreatic tumors occurred apart from the surgical margin; thus, the first mechanism is highly unlikely. ITPN is an intraductal tumor, and intraductal proliferation reportedly appears to extend from the MPD into the smaller secondary ducts in many cases of ITPN [7]. If the remnant pancreatic tumor develops in a metachronous, multicentric fashion, the tumor is likely to be located mainly in the MPD; in the present case, however, the pancreatic tail lesion was located far from the MPD. In addition, the molecular alterations between the original and recurrent lesion are likely to differ from each other in a metachronous, multicentric manner; in the present case, however, all molecular features were identical. Therefore, it is reasonable to think that remnant pancreatic tumors originate from intraductal or intrapancreatic lymphovascular spread of the initial tumor rather than by metachronous, multicentric development. Although it is difficult to determine the true mechanism, we speculate that the remnant pancreatic tumors in the present case were most likely recurrences of the original lesion.

## Conclusions

We experienced a case of a recurrent ITPN with an invasive component after a 16-year interval from the initial surgery of a noninvasive ITPN. Because ITPN is a recently established entity and most of its biological nature remains unclear, long-term postoperative surveillance is recommended even after surgery for noninvasive ITPN.

## Abbreviations

ACC: Acinar cell carcinoma
CK: Cytokeratin
CT: Computed tomography
IPMN: Intraductal papillary mucinous neoplasm
ITPN: Intraductal tubulopapillary neoplasm
MPD: Main pancreatic duct
PanIN: Pancreatic intraepithelial neoplasm
PDAC: Pancreatic ductal adenocarcinoma
WHO: World Health Organization
